# Energy security and its determinants in New Zealand

**DOI:** 10.1007/s11356-024-34611-0

**Published:** 2024-08-08

**Authors:** Saeed Solaymani

**Affiliations:** Scion (New Zealand Forest Research Institute Ltd), Titokorangi Drive (Formerly Longmile Road), Rotorua, 3046 New Zealand

**Keywords:** Global oil prices, Energy security, Fossil fuel consumption, Energy efficiency, Simulation

## Abstract

New Zealand relies on imported fossil fuels for about 38% of its primary energy. The country’s energy demand is expected to grow due to population and economic growth, which will put more pressure on the energy system. Besides, resource scarcity, energy price volatility, and environmental challenges have made energy security a major concern for New Zealand and other countries. Given the lack of significant research on the effects of energy security factors in New Zealand, this study aims to shed light on the primary determinants of energy security using the dynamic autoregressive distributed lag method based on time series data from 1978 to 2021. The study found that a long-run link exists between energy security and energy intensity (energy efficiency), renewable energy use, fossil fuel consumption, and global oil prices. Real GDP, renewable energy consumption, and energy security were found to improve energy security, while fossil fuel consumption and world oil prices had a negative impact. The study also revealed a one-way causality from real GDP, fossil fuel consumption, and renewable energy use to energy security. In contrast, the relationship between energy intensity and energy security is bidirectional. Simulation results showed that global crude oil prices have a lower impact on energy security compared to other variables and are most responsive to a 5% shock in fossil fuel consumption, followed by economic growth.

## Introduction

Energy can be obtained from many or several sources. A lower number of energy sources increases the risk of energy availability. More local production of various energy resources leads to more availability and access to energy. Finally, importing energy from multiple countries with high political stability reduces risks to energy availability (Bhattacharyya 2011). All these subjects show the importance of energy security. High dependency on external energy sources for European countries makes it difficult to be protected from external energy shocks (Mišík, 2022). Energy security is an economic, environmental, political, and social phenomenon in the literature that is all interconnected simultaneously. Evidence highlights that energy security has many dimensions, including climatic, economic, and international factors (Kuceba et al. 2010; Hipple et al. 2011). High global oil prices affect fossil fuel consumption, which increases concern about the vulnerability, affordability, and reliability of energy. Energy security has different effects on income distribution, depending on the stage of economic development. In the early stage, it has a negative impact, but after a certain level of development, it improves income inequality (Lee et al. 2022). Energy security has a vital role in increasing renewable energy production and consumption and nuclear energy production in many countries (Zaman 2015; Chu et al. 2023). Evidence shows that wind, green hydrogen, hydroelectricity, and other renewable energies can increase energy security (Xiang et al. 2021; Cergibozan 2022).

New Zealand’s total primary energy supply in 2021 was 872.18 petajoules (PJ), of which coal provided 7.5%, oil 34%, natural gas 17.6%, and renewable energies 40.8%, and the remaining 0.1% came from waste heat. The country imports about 38.1% of its total primary energy, which includes coal (11.6% of total imports), oil (88.4%), and renewables (0.04%) (MBIE 2023). The major consumer of this amount of imported energy is transportation. A change in global oil prices has a direct impact on the prices of imported energy, leading to fluctuations in transportation costs. This, in turn, has a significant impact on the country’s energy security. This is because the availability of energy sources with affordable prices, i.e., energy security, will decrease (IEA 2022). Nearly three-quarters, or around 72%, of the country’s primary energy is generated through domestic sources such as electricity, gas, and coal. However, it must be noted that the country’s reliance on imported energy products remains considerably high. The insecurity in energy resources has increased in recent years because of the increases in global crude oil prices, Chinese military movements near Taiwan’s borders, and Russia’s invasion of Ukraine. As the population and economic growth continue to increase, the demand for both fossil fuels and renewable energies in New Zealand is set to increase. Therefore, it is necessary to investigate the impacts of different variables on New Zealand’s energy security, as it is a multidimensional phenomenon influenced by changes in economic, energy, and environmental variables.

Considering the unique risks posed by global crude oil prices and other domestic factors to energy security and the limited coverage of this topic in existing literature, this study focuses on examining the primary factors influencing energy security in New Zealand. This study estimates the long-run relationship between energy security and several explanatory variables, such as GDP, energy intensity, crude oil prices, renewable energy consumption, and fossil fuel consumption, by applying the autoregressive distributed lag (ARDL) bounds test method. It then undertakes dynamic ARDL simulations to confirm the effects of the changes in economic growth, renewable and fossil fuel consumption, and global oil prices on energy security. Besides the insufficient amount of evidence to support the inclusion of the proposed variables in the existing literature, this study specifically focuses on the impacts of crude oil prices and energy intensity on energy security. Therefore, the motivation behind this investigation, as well as the appropriate empirical methods employed, seems to be valuable contributions to the existing body of knowledge. The results of this paper will help energy policymakers understand the role of key economic and energy variables in the energy security of New Zealand.

The rest of the paper is structured as follows: the “Literature review” section offers a survey of the literature. In the “Data and method” section, data and methodology are discussed. The “Results and discussion” section explains the empirical results, and the “Conclusions and policy implications” section concludes the outcomes and suggests policy measures.

## Literature review

As discussed above, energy security is strongly related to national security. Some studies believe that it is not only a national concern but also an international concern (Mara 2022). To achieve an appropriate level of energy security, countries need to achieve all dimensions of energy security, such as availability, applicability, acceptability, and affordability (Amin et al. 2022). But other studies discussed these dimensions from three perspectives, i.e., sovereignty, resilience, and robustness (Rodríguez-Fernández et al. 2022).

There are a lot of different variables and indices for measuring energy security. For example, many studies used energy intensity as a proxy for energy security (Yao and Chang 2014; Sotnyk et al. 2021; Nasir et al. 2022). Other studies used the share of non-fossil consumption in the country’s total energy supply (Fang et al. 2018; Sotnyk et al. 2021) and net energy imports (Lin and Raza 2020). Lu et al. (2019) and Zhang et al. (2021) used a combination of several indices, such as energy availability, affordability, energy efficiency, and environmental impact, to measure energy security. In terms of methodology in this context, researchers have used various methods for estimating the effects of different variables on energy security in the literature. For example, Taghizadeh-Hesary et al. (2022) used the generalized method of moments (GMM) to investigate the impact of energy security on poverty. Nasir et al. (2022) used a Tobit model and found an interconnection between energy security and poverty. Gasser (2020) also used 63 indices in multiple dimensions and showed that there is no transparency in the selection of energy security indicators. Shah et al. (2019) used mathematical programming to analyze energy security in South Asia. Other studies showed that energy intensity in some years reduces energy security in European countries, but when this variable is used with per capita GDP and carbon intensity, it has the highest impact on energy security (Radovanović et al. 2017).

Different variables have various impacts on energy security. For example, high fossil fuel consumption and energy imports increase energy insecurity in African countries (Alemzero et al. 2021). Le and Nguyen (2019) demonstrated that energy security can lead to economic growth, while the energy insecurity index, calculated from energy intensity and carbon intensity, has a negative effect on economic growth. The improvement of energy efficiency and the regulatory framework for energy saving can develop the use of bioenergy resources and increase energy security (Dźwigoł et al. 2019).

However, low energy technology efficiency cannot enhance energy security (Ainou et al. 2022). Fu et al. (2021) believe that increasing energy prices can reduce energy demand, while expanding the options of available energy sources can improve energy security. Other studies showed that high energy prices reduce the affordability of energy and reduce energy security (Hipple et al. 2011). To increase energy security and environmental sustainability through green energy resources, it is essential to implement some financial policies such as green bonds, green central banking, and carbon market instruments (Sachs et al. 2019). Brodny and Tutak (2021), based on an entropy method, showed that in terms of energy security, the Czech Republic has a high level and Poland has the lowest among the four new European countries. Wang et al. (2022), using a cross-sectional autoregressive lag model, also showed that globalization and financial development reduce energy security, and technological development increases it. They also found that energy intensity, per capita GDP, and carbon intensity impact energy security the most. A move toward renewable energy production in European countries has increased their energy security (Gökgöz and Güvercin 2018).

The literature above indicates that numerous studies on energy security evaluation rely on constructing indices, while only a few are on econometric analyses. While many studies have examined the influence of energy security on GDP or economic growth, surprisingly, no research has yet explored the impact of economic growth on energy security. Therefore, this study attempts to improve the literature by analyzing the impacts of various variables (economic growth, energy imports and exports, and renewable and non-renewable energy use) on energy security in one of the Asia–Pacific countries, i.e., New Zealand, using several econometric methods.

### Data and method

We use annual data for the period from 1978 to 2021. GDP data was collected from Stats NZ; energy data was collected from the Ministry of Business, Innovation, and Employment (MBIE); and global oil prices were collected from the OPEC website. The data for the financial development index was collected from the International Monetary Fund database.

Many studies have evaluated the impacts of different variables on energy security. Fu et al. (2021) discovered that energy prices and energy security are positively correlated. However, Hipple et al. (2011) found a contradictory result, showing a negative relationship. Renewable energy consumption also improves energy security and decreases the need for energy imports (Gökgöz and Güvercin 2018). Energy imports also increase countries’ challenges and decrease their energy security (Radovanović et al. 2017). High fossil fuel consumption and energy imports increase energy insecurity (Alemzero et al. 2021). Le and Nguyen (2019) demonstrated that energy security can enhance economic growth. In their study, Radovanović et al. (2017) demonstrated that energy security, when considered together with per capita GDP and carbon intensity, emerges as the most influential factor in determining energy security. According to Feng et al. (2023), financial development can affect energy security either positively or negatively, depending on the economic situation of the country.

In this study, according to Bhattacharyya (2011), we define energy security (ESEC) as a function of economic growth (real GDP = RGDP), renewable energy consumption (RCON), fossil fuel consumption (FCON), energy intensity (ENINT), global oil prices (WOP), and financial development (FINDEV). The consumption of fossil fuels and renewable energy instead of production was selected to reduce the multicollinearity in the model. Energy data is based on petajoules, GDP is based on the New Zealand dollar, and global oil prices are based on the US dollar per barrel. The general equation of the study model is as follows:

1 $$\text{ESEC}=\text{f }(\text{RGDP},\text{ RCON},\text{ FCON},\text{ ENINT},\text{ WOP},\text{ FINDEV})$$

In Eq. (1), according to Bhattacharyya (2011), energy security can be defined as the following formula:

2 $$\text{Energy security }(\text{ESEC})=\frac{\text{Energy imports}-\text{Energy exports}}{\text{Total primary energy supply}}$$

Following Nair et al.’s (2021) study, we use the ratio of total renewable energy production to total energy supply instead of energy security index in Eq. (2) to ensure the reliability of the results of the study.

We can linearize and rewrite Eq. (1) to Eq. (3) below by transforming the variables into their natural logarithmic values.

3 $$\text{ESEC}={ \rho }_{0}+{\rho }_{1}{\text{RGDP}}_{\text{t}}+{ \rho }_{2}{\text{RCON}}_{t}+{ \rho }_{3}{\text{FCON}}_{t}+{ \rho }_{4}{\text{ENINT}}_{t}+{ \rho }_{5}{\text{WOP}}_{t}+{ \rho }_{6}{\text{FINDEV}}_{t}+{u}_{t}$$

where *ρ*_0_, …, *ρ*_6_ are the coefficients of Eq. (3). Based on the literature, it is expected that the signs of RGDP, ENINT, and RECON are positive and the signs of FCON and WOP are negative, while the signs of FINDEV depend on the economic condition of the country, which may be positive or negative.

### The ARDL approach

To investigate the short- and long-run impacts of various indicators on energy security, we apply an autoregressive distributed lag (ARDL) model, which is formulated based on the variables under consideration as below.

4 $$\Delta {\text{ESEC}}_{t}={\psi }_{0}+{\psi }_{1}{\text{ESEC}}_{t-1}+{\psi }_{2}{\text{RGDP}}_{t-1}+{\psi }_{3}{\text{RCON}}_{t-1}+{\psi }_{4}{\text{FCON}}_{t-1}+{\psi }_{5}{\text{ENINT}}_{t-1}++{\psi }_{6}{\text{WOP}}_{t-1}+{\psi }_{7}{\text{FINDEV}}_{t-1}+\sum\nolimits_{i=1}^{l}{\phi }_{1}\Delta {\text{ESEC}}_{t-i}+\sum\nolimits_{i=1}^{l}{\phi }_{2}\Delta {\text{RGDP}}_{t-i}+\sum\nolimits_{i=1}^{l}{\phi }_{3}\Delta {\text{RCON}}_{t-i}+\sum\nolimits_{i=1}^{l}{\phi }_{4}\Delta {\text{FCON}}_{t-i}+\sum\nolimits_{i=1}^{l}{\phi }_{5}\Delta {\text{ENINT}}_{t-i}+\sum\nolimits_{i=1}^{l}{\phi }_{6}\Delta {\text{WOP}}_{t-i}+\sum\nolimits_{i=1}^{l}{\phi }_{7}\Delta {\text{FINDEV}}_{t-i}+{u}_{t}$$

In Eq. (4), all variables are in their logarithmic forms. The parameters $\psi$ and $\phi$ are the long- and short-run coefficients of the model that will be estimated, and *u* and *l* are the error term and optimal lag length, respectively. The ARDL model is a powerful tool that facilitates the estimation of a wide range of variables with different levels of stationary. It is also a suitable estimator for small sample models. The null hypothesis for the ARDL estimation using the *F*-statistics suggests that there is no long-run cointegration (${H}_{0}:{\psi }_{1}={\psi }_{2}={\psi }_{3}={\psi }_{4}={\psi }_{5}={\psi }_{6}={\psi }_{7}=0$), while the alternative states that there is. The estimated *F*-statistics will be compared with the criteria proposed by Pesaran et al. (2001).

### The dynamic ARDL method

In the ARDL methodology, there was a limitation of the user not being able to simulate the impact of a change in one of the independent variables on the dependent variable. To solve this problem, Jordan and Philips (2018) proposed a method known as dynamic simulations of the ARDL model. The method first estimates using the ordinary least squares (OLS) technique. Then, it simulates a multivariate normal distribution based on the specified number of simulations for the parameters vector, which is 5000 in this study. There are a lot of studies that have applied the methodology, such as Solaymani (2023), Das et al. (2022), Ali et al. (2022), and Hossain et al. (2022). This method uses Eq. (3) to yield Eq. (5).

5 $$\Delta {\text{ESEC}}_{t}={\psi }_{0}+{\psi }_{1}{\Delta \text{RGDP}}_{t}+{\psi }_{2}{\Delta \text{RCON}}_{t}+{\psi }_{3}\Delta {\text{FCON}}_{t}+{\psi }_{4}{\Delta \text{ENINT}}_{t}+{\psi }_{5}\Delta {\text{WOP}}_{t}+{\psi }_{6}\Delta {\text{FINDEV}}_{t}+{\phi }_{1}{\text{RGDP}}_{t-1}+{\phi }_{2}{\text{RCON}}_{t-1}+{\phi }_{3}{\text{FCON}}_{t-1}+{\phi }_{4}{\text{ENINT}}_{t-1}+{\phi }_{5}{\text{WOP}}_{t-1}+{\phi }_{6}{\text{FINDEV}}_{t-1}+{u}_{t}$$

In Eq. (5), the definition of parameters,$\psi$ and $\phi$, is the same as Eq. (5). In addition to introducing a new variable for energy security, we employ a vector autoregression (VAR) model to verify the robustness of the ARDL model’s results.

## Results and discussion

The results of the descriptive statistics for all variables under investigation are presented in Table 1.

**Table 1:** Results for the descriptive statistics

	ESEC	ENINT	RCON	FCON	WOP	RGDP	FINDEV
Mean	3.155	1.036	3.332	5.944	3.470	1.732	1.641
Median	3.098	1.031	3.445	6.081	3.363	1.764	1.457
Maximum	3.852	1.125	3.726	6.223	4.631	3.080	5.587
Minimum	2.667	0.691	2.807	5.399	2.491	0.010	− 3.812
Std. Dev	0.320	0.150	0.320	0.255	0.628	0.739	1.795
Skewness	0.377	− 0.253	− 0.335	− 0.863	0.390	− 0.397	− 0.35
Kurtosis	2.345	2.312	1.500	2.363	1.971	2.851	4.434
Jarque–Bera	1.784	1.338	4.953	6.202	2.985	1.171	4.668
Probability	0.410	0.512	0.084	0.045	0.225	0.557	0.097

We used the ADF and PP tests to determine the stationarity of the variables in the model (Table 2). We performed these tests with intercept and with intercept and trend. Results show that except for financial development (FINDEV), all variables are stationary at their first differences, and, therefore, they are in order of 1, *I*(1). This condition allows us to use the ARDL model to examine the objectives of the study.

**Table 2:** The results for the stationary test

Test	Include		ESEC	ENINT	RCON	FCON	WOP	RGDP	FINDEV
ADF	Int	*I*(0)	− 2.165	− 0.659	− 0.551	− 2.514	− 1.582	− 0.651	− 4.723^e^
		*I*(1)	− 7.507^e^	− 2.766^e^	− 7.572^e^	− 7.758^e^	− 6.278^e^	− 4.786^e^	− 7.988^e^
	T&Int	*I*(0)	− 2.057	− 1.593	− 2.421	− 1.089	− 2.065	− 2.464	− 4.740^e^
		*I*(1)	− 7.975^e^	− 3.168	− 7.478^e^	− 8.398^e^	− 6.150^e^	− 4.716^e^	− 7.883^e^
PP	Int	*I*(0)	− 2.150	− 0.159	− 0.500	− 1.436	− 1.603	− 0.848	− 4945^e^
		*I*(1)	− 7.425^e^	− 7.312^c^	− 7.583^e^	− 7.731^e^	− 6.283^e^	− 4.786	− 12.256^e^
	T&Int	*I*(0)	− 2.057	− 1.046	− 2.460	− 1.005	− 2.131	− 2.076	− 4.934^e^
		*I*(1)	− 7.980^e^	− 7.613^c^	− 7.486^e^	− 9.343^e^	− 6.141^e^	− 4.716^e^	− 12.083^e^

^c,d,e^Significant levels at 10%, 5%, and 1%, respectively

**Fig. 1:**
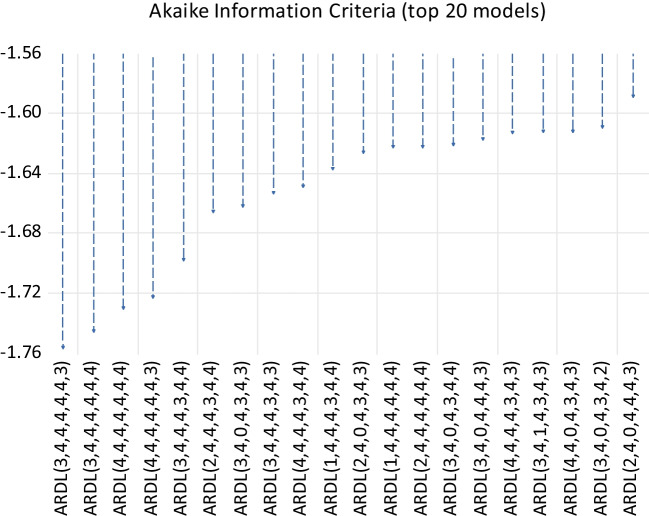
Optimal lag length selection

Figure 1 shows the number of lags that were selected for the ARDL model using the Akaike information criteria. It shows that the optimal lag length is 4 for the model.

### ARDL results

Next, we need to find out whether there is a long-run association between model variables or not. To do this test, we use the *F*-bounds test, which is a joint test. The null hypothesis of this test checks for long-run relationships between model variables and the alternative check-investment condition. Table 3 reports the bounds test results and shows that the null hypothesis is rejected, indicating that a significant long-run relationship between the model variables at a 5% level of significance.

**Table 3:** *F*-bounds test

Test statistic	Value					
*F*-statistic	4.435					
Critical values	10%		5%		1%	
Sample size	*I*(0)	*I*(1)	*I*(0)	*I*(1)	*I*(0)	*I*(1)
35	2.254	3.388	2.685	3.96	3.713	5.326
40	2.218	3.314	2.618	3.863	3.505	5.121
Asymptotic	1.99	2.94	2.27	3.28	2.88	3.99

The long-run results of the ARDL model in Table 4 show that economic growth has a positive impact on energy security, and the 1% increase in economic growth increases energy security by 6.77%. In the short run, economic growth and energy security have a positive relationship, with a 1% growth leading to a 7.55% increase in energy security. This is because a large percentage of energy supply in New Zealand is supplied by domestic energy sources (57%), particularly electricity. As the economy grows, the use of more renewable energy sources improves energy security in the country. Therefore, a high level of energy security, with diverse sources of energy, enables a country to grow more effortlessly. Recent research by Ayaz et al. (2024) reveals a positive relationship between energy security and economic growth. Renewable energy consumption improves energy security in the short and long run, as the short- and long-run coefficients of RCON are positive and statistically significant. They show that if renewable energy consumption increases by 1%, energy security increases by 3.39% and 2.08% in the long and short run, respectively. This is because having more energy sources in the country enhances its energy security. Gökgöz and Güvercin (2018) found in their study that renewable energy consumption improves energy security. The coefficients of fossil fuel consumption are negative in the short and long run and are significant at a 1% significance level. Its coefficients show that increasing fossil fuel consumption by 1% reduces long-run energy security by 10.47% and short-run energy security by 3.15%. The rise in fossil fuel consumption and energy imports has heightened energy insecurity in African nations (Alemzero et al. 2021).

**Table 4:** The ARDL (3, 4, 4, 4, 4, 4, 3) error correction results

Variable	Coeff [Std. err.]	*t*-stats [Prob.]	Variable	Coeff [Std. err.]	*t*-stats [Prob.]
ESEC(− 1)	− 1.017^d^ [0.359]	− 2.830 [0.025]	*D*(RCON(− 2))	− 1.718^e^ [0.585]	− 2.935 [0.011]
RGDP(− 1)	6.773^e^ [1.844]	3.673 [0.008]	*D*(RCON(− 3))	− 2.730^e^ [0.643]	− 4.244 [0.001]
RCON(− 1)	3.389^d^ [1.466]	2.311 [0.054]	*D*(FCON)	− 3.146^e^ [0.600]	− 5.243 [0.000]
FCON(− 1)	− 10.471^c^ [2.174]	− 4.817 [0.002]	*D*(FCON(− 1))	6.920^e^ 1.080]	6.410 [0.000]
ENINT(− 1)	4.894^c^ [2.318]	2.111 [0.073]	*D*(FCON(− 2))	5.052^e^ [0.770]	6.559 [0.000]
WOP(− 1)	− 0.991^d^ [0.412]	− 2.405 [0.047]	*D*(FCON(− 3))	2.781^e^ [0.704]	3.950 [0.001]
FINDEV(− 1)	− 0.099^c^ [0.049]	− 2.024 [0.083]	*D*(ENINT)	0.859 [0.580]	1.482 [0.160]
Cons	− 28.236 15.309]	− 1.844 [0.108]	*D*(ENINT(− 1))	− 4.499^e^ [0.804]	− 5.598 [0.000]
**ECT(− 1)**	** − 1.017**^**e**^** [0.121]**	** − 8.424 [0.000]**	*D*(ENINT(− 2))	− 3.367^e^ [0.836]	− 4.028 [0.001]
D(ESEC(− 1))	0.150 [0.119]	1.257 [0.229]	*D*(ENINT(− 3))	2.241^e^ [0.760]	2.949 [0.011]
D(ESEC(− 2))	0.381^e^ [0.110]	3.454 [0.004]	*D*(WOP)	− 0.036 [0.078]	− 0.460 [0.652]
*D*(RGDP)	7.555^e^ [1.612]	4.685 [0.000]	*D*(WOP(− 1))	0.648^e^ [0.119]	5.445 [0.000]
*D*(RGDP(− 1))	− 0.502 [1.735]	− 0.290 [0.776]	*D*(WOP(− 2))	0.626^e^ [0.097]	6.484 [0.000]
*D*(RGDP(− 2))	2.147 [1.655]	1.297 [0.216]	*D*(WOP(− 3))	0.323^e^ [0.071]	4.576 [0.000]
*D*(RGDP(− 3))	− 5.512^e^ [1.281]	− 4.305 [0.001]	*D*(FINDEV)	− 0.056^e^ [0.011]	− 4.964 [0.000]
*D*(RCON)	2.079^e^ [0.442]	4.702 [0.000]	*D*(FINDEV(− 1))	0.015 [0.009]	1.657 [0.120]
*D*(RCON(− 1))	− 3.672^e^ [0.783]	− 4.689 [0.000]	*D*(FINDEV(− 2))	0.027^e^ [0.009]	3.053 [0.009]
Diagnostic tests					
Statistic	Value [Prob.]	Test	Statistic	Value [Prob.]	Test
Adj. *R*-squared	0.804		ARCH *F*(1,37)	0.002 [0. 965]	Heteroskedasticity
Jarque–Bera	0.424 [0.809]	Normality	Ramsey RESET	0.398 [0.704]	Model specification
Breusch-Godfrey	2.424 [0.171]	Serial correlation	Durbin–Watson	2.533	

^c,d,e^Significant levels at 10%, 5%, and 1%, respectively

Energy intensity as a proxy for energy efficiency also improves energy security in both the short and long run. This is because evidence shows that the energy intensity of the country has decreased over time and shows improvements in energy efficiency. A 1% increase in energy intensity increases energy security by 4.89% and 0.86% in the short and long run, respectively, while the short-run coefficient is insignificant. The former is because an improvement in energy efficiency may not immediately reduce energy consumption in the country. The effects will be noticeable in the long run. Radovanović et al. (2017) showed that energy security can help improve energy security in European countries. The global oil price has a negative coefficient in both the short and long run, but the coefficient is not statistically significant in the short run. It shows that when global prices of crude oil increase, it becomes evident that the cost of importing crude oil and petroleum products for the country also increases. This, in turn, leads to alarming increase in the country’s energy insecurity. Hipple et al. (2011) demonstrated that high energy prices not only make energy less affordable but also compromise energy security. This vulnerability underscores the need for diversified energy sources and an effective energy security strategy. The small and insignificant coefficient of WOP may be because countries buy crude oil through long-term contracts. The coefficient of financial development is negative and statistically significant in both the short and long run. It shows that when financial development increases by 1%, energy security in the country decreases by 0.1% and 0.06% in the short and long run, respectively. Feng et al. (2023) showed in their study of 60 countries that financial developments had a positive impact on energy security during 2010–2015 but had a negative effect during 2013–2017.

The results of long-run cointegration in Table 5 confirm the log-run results of the error correction model in Table 5. Therefore, we can conclude that a positive long-run cointegration relationship exists between RGDP, RCON, and ENINT and energy security. However, a negative long-run cointegration relationship occurred between FCON and ESEC.

**Table 5:** Cointegrating coefficients

Variable	Coeff	Std. error	*t*-statistic	Prob
RGDP(− 1)	6.658	3.262	2.041	0.049
RCON(− 1)	3.332	1.087	3.066	0.004
FCON(− 1)	− 10.293	3.648	− 2.822	0.008
ENINT(− 1)	4.811	3.438	1.400	0.171
WOP	− 0.974	0.288	− 3.380	0.002
FINDEV(− 1)	− 0.097	0.045	− 2.145	0.039
*C*	− 27.757	22.034	− 1.260	0.217

According to the coefficient of error correction term (ECT(− 1)) variable, the short-run speed of achieving a long-run equilibrium due to a change in the model is 102% in each period. The results of the diagnostic tests are presented in Table 4, indicating that the ARDL model successfully passes all diagnostic tests, particularly due to the absence of serial correlation among model variables.

**Fig. 2:**
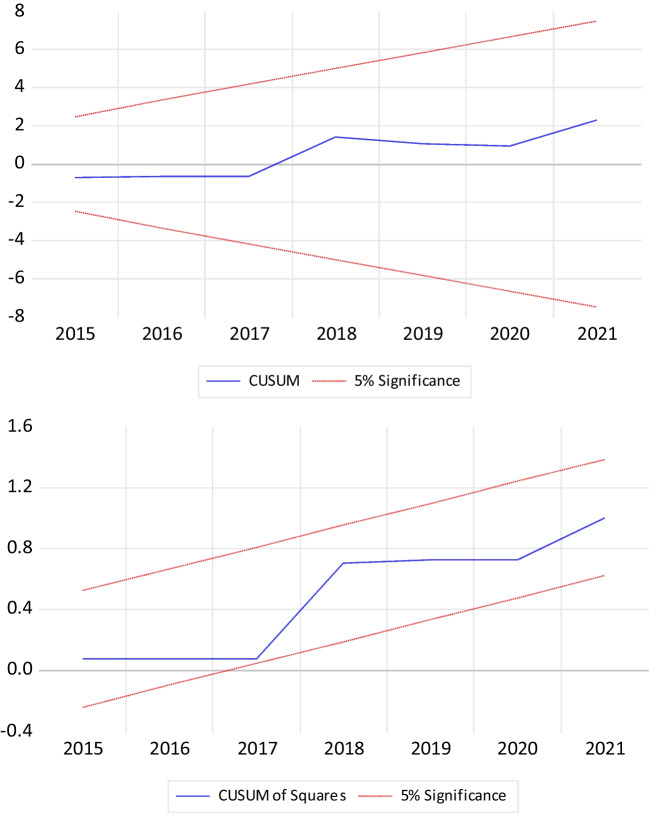
Model stability tests

To check the parameter stability, we use the cumulative sum (CUSUM) and cumulative sum of squares (CUSUM) tests proposed by Brown et al. (1975). The results of these tests are provided in Fig. 2, implying that the estimation of the parameters of the model is stable.

Different methods can be used to check the robustness of the results of the ARDL model, such as dynamic OLS and fully modified OLS. In this study, we used another index for energy security, i.e., the ratio of renewable energy production to total energy supply, to check the validity of the results of the main model. The results of this model are provided in Table 10 in the Appendix. The results of this model confirm the results of the main model of the study. We also used the Bayesian vector autoregression (VAR) with the Minnesota prior type to check the robustness of the results. The results of this estimator are presented in the Appendix (Table 11). Based on the Akaike criteria for lag selection in the VAR model, the number of optimal lags for this study is one, VAR(3) (Table 6).

**Table 6:** VAR lag length selection

Lag	LogL	LR	FPE	AIC	SC	HQ
0	64.37906	NA	1.44E − 10	− 2.79898	− 2.50642	− 2.69244
1	332.3303	431.3362	3.42E − 15	− 13.4795	− 11.13904*	− 12.62725*
2	390.3378	73.57040*	2.73e − 15*	− 13.9189	− 9.5305	− 12.3209
3	451.5626	56.74501	2.82E − 15	− 14.51525*	− 8.07891	− 12.1715

**Fig. 3:**
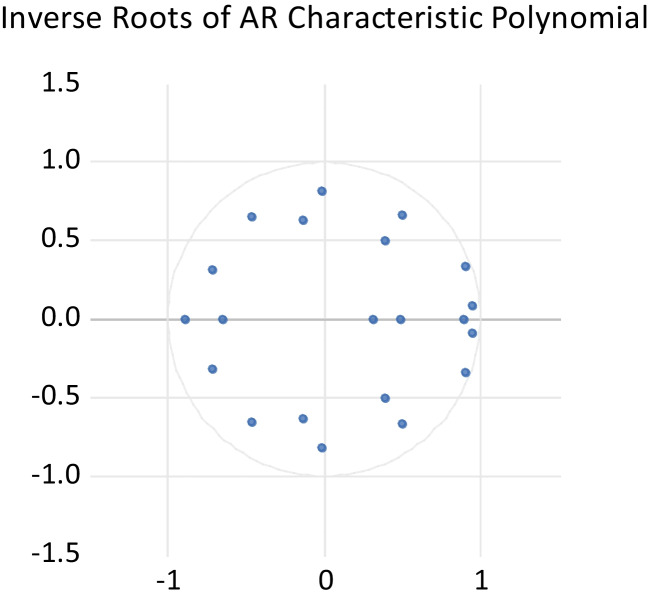
Model stability test

To examine the stability of the VAR(3), we used the inverse root of the AR characteristic polynomial, which is presented in Fig. 3. Since all the inverse roots of VAR(3) are located within the unit circle, the VAR model is stable. The autocorrelation test was performed using the LM serial correlation in our Bayesian VAR. The result of this test is provided in Table 7, implying that there is no evidence of serial correlation in the model.

**Table 7:** LM test results for serial correlation of residual VAR(3)

	At lag *h*		At lags 1 to *h*	
Lag	Rao *F*-sta	Prob	Rao *F*-sta	Prob
1	5.574	0.000	5.574	0.000
2	7.607	0.000	206.816	0.001
3	5.480	0.000	NA	NA

### The causality results

To check the causality between each independent variable and energy security and vice versa, we use the VAR causality test (Table 8). The results of the Granger causality test for a pair of variables show that a one-way causality link exists from RGDP to ESEC. It means that economic growth improves energy security because of the high production of domestic renewable energy sources in the country that help economic growth. Another unidirectional causality was established from renewable energy consumption to energy security and from fossil fuel consumption to ESEC. This suggests that both types of energy resources impact energy security, rather than energy security influencing them. A one-way causality link happens from RCON to RGDP and from FCON to RGDP showing the growth hypothesis. According to Soytas and Sari (2003), Lee (2006), Ewing et al. (2007), Payne (2010), and Das et al. (2022), if a one-way causality link occurs from energy use to economic growth, we have a growth hypothesis. In this condition, a reduction in energy use negatively affects economic growth because the growth of the country will not benefit from its main input. However, the inverse relationship establishes the conservation hypothesis. Chang and Fang (2022) also found a growth hypothesis for the connection between renewable energy use and economic growth in BRICS countries.

**Table 8:** Granger causality results based on VAR

	ESEC	RGDP	RCON	FCON	ENINT	WOP	FINDEV
ESEC		4.010 [0.135]	1.689 [0.430]	3.547 [0.170]	4.677 [0.096]^e^	1.844 [0.398]	7.883 [0.048]^d^
RGDP	14.698 [0.001]^c^		4.875 [0.087]^e^	8.409 [0.015]^d^	1.750 [0.417]	7.246 [0.027]^d^	2.640 [0.450]
RCON	6.132 [0.047]^d^	2.896 [0.231]		16.983 [0.000]^c^	0.474 [0.789]	1.150 [0.563]	1.821 [0.610]
FCON	6.983 [0.030]^d^	5.899 [0.052]^d^	3.697 [0.157]		2.34 [0.310]	0.156 [0.925]	2.840 [0.417]
ENINT	24.204 [0.000]^bc^	5.141 [0.076]^e^	9.213 [0.010]^c^	1.264 [0.532]		5.881 [0.053]^d^	4.569 [0.206]
WOP	2.862 [0.239]	1.692 [0.429]	16.501 [0.000]^c^	10.04 [0.007]^c^	4.430 [0.109]		6.840 [0.077]^c^
FINDEV	10.084 [0.018]^d^	2.677 [0.444]	14.720 [0.002]^e^	0.985 [0.805]	0.398 [0.940]	0.443 [0.931]	

^c,d,e^Significant levels at 10%, 5%, and 1%, respectively

Other unidirectional relationships accrued from ENINT to RGDP, from FCON to RCON, from ENINT to RCON, from WOP to RCON, from WOP to FCON, and from WOP to ENINT. Finally, a bidirectional relationship exists between ENINT and ESEC, between FCON and RGDP, and between financial development and energy security.

### Results for the dynamic ARDL simulations

The results of the dynamic ARDL simulation model are reported in Table 9. These results indicate that the short-run speed of the model to achieve long-run equilibrium is 42%. In the short run, the coefficients of the real GDP, RCON, and FCON variables are significant and have the expected signs. In the short run, real GDP plays a primary role in ensuring energy security followed by RCON and FCON. The long-run coefficients, similar to the main ARDL model, have the expected signs, except for FINDEV, and are significant at appropriate significance levels. Similar to the short-run results, in the long run, FCON is one of the main contributors to reducing energy security, while energy intensity and real GDP contribute positively. These results confirm the actual results of the country that energy intensity (energy efficiency), along with a significant amount of domestic production of renewable energy, helps economic growth. The impact of global oil prices is not high in both the short and long run. It alerts energy policymakers to focus on other important factors that improve energy security such as renewable energies and reduce their dependency on fossil fuel imports. These findings also help them to understand the importance of different variables on energy security to prepare adequate policies to reduce the negative impact of sudden increases in global oil prices on domestic markets.

**Table 9:** Dynamic ARDL simulations results

ΔESEC	Coefficient	Std. err	*t* [*P* > *t*]
ECT(− 1)	− 0.417^e^	0.135	− 3.1 [0.004]
L2_RGDP	1.249^c^	0.733	1.71 [0.099]
L2_RCON	0.858	0.587	1.46 [0.155]
L2_FCON	− 2.211^d^	1.004	− 2.2 [0.036]
L2_ENINT	0.684	1.018	0.67 [0.507]
L1_WOP	− 0.122	0.131	− 0.93 [0.36]
L2_FINDEV	0.012	0.016	0.73 [0.474]
D_RGDP	4.507^e^	1.724	2.61 [0.014]
D_RCON	1.978^d^	0.863	2.29 [0.03]
D_FCON	− 1.591^c^	0.819	− 1.94 [0.062]
D_ENINT	− 1.009	1.15	− 0.88 [0.388]
D_WOP	0.062	0.115	0.54 [0.594]
D_FINDEV	− 0.004	0.012	− 0.33 [0.747]
_CONS	− 3.667	6.168	− 0.59 [0.557]
*F*(11, 30)	13.73	Prob > *F*	0.06
*R*-sq	0.462	Adj *R*-sq	0.217
N	42	Simulations	5000

^c,d,e^Significant levels at 10%, 5%, and 1%, respectively

**Fig. 4:**
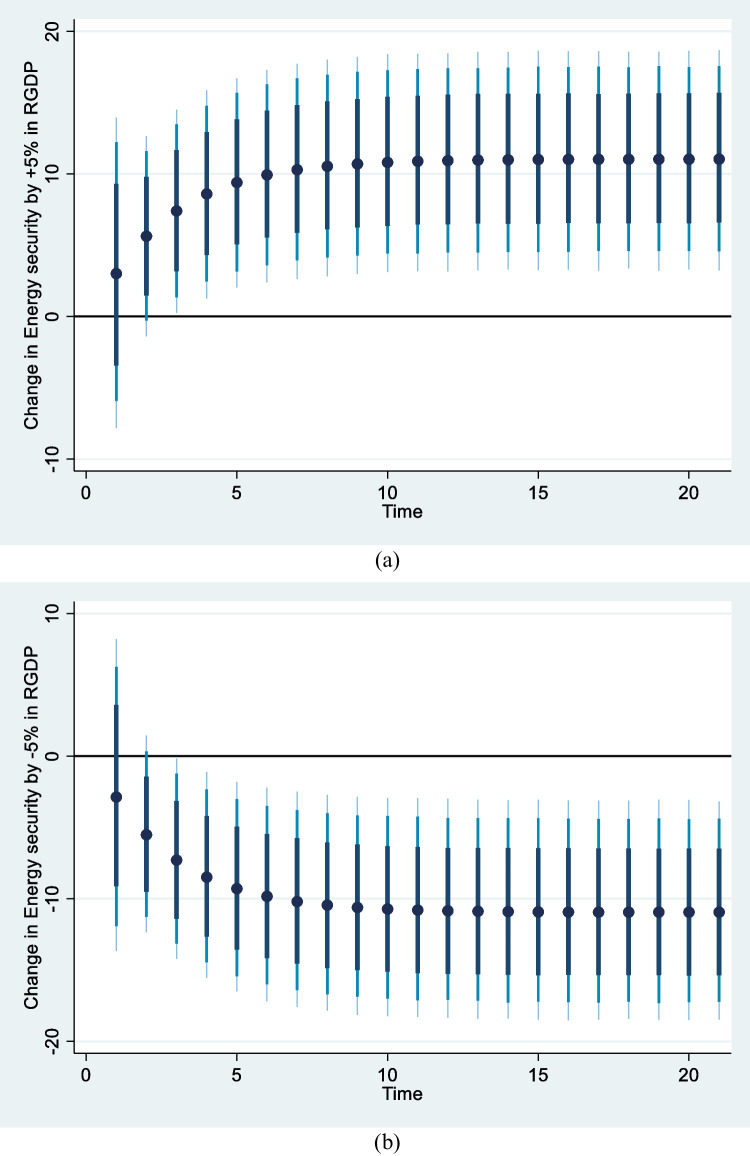
Response of energy security to a 5% **a** increase and **b** decrease in economic growth

**Fig. 5:**
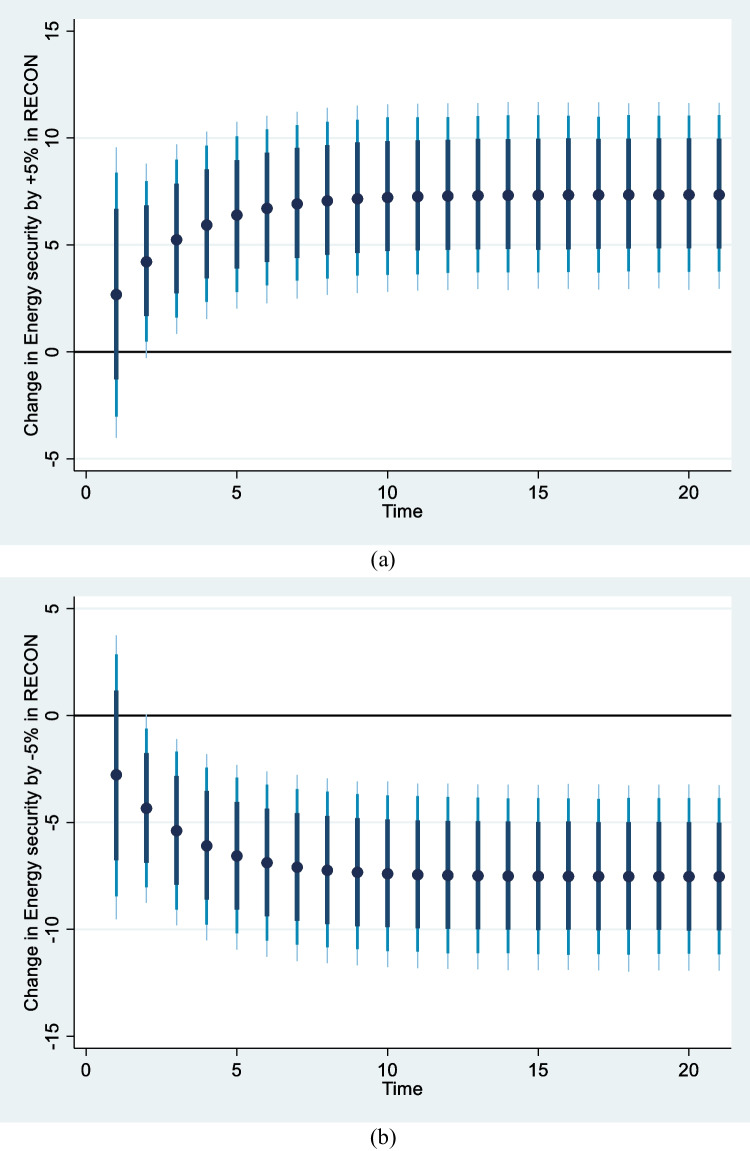
Response of energy security to a 5% **a** increase and **b** decrease in renewable energy consumption

**Fig. 6:**
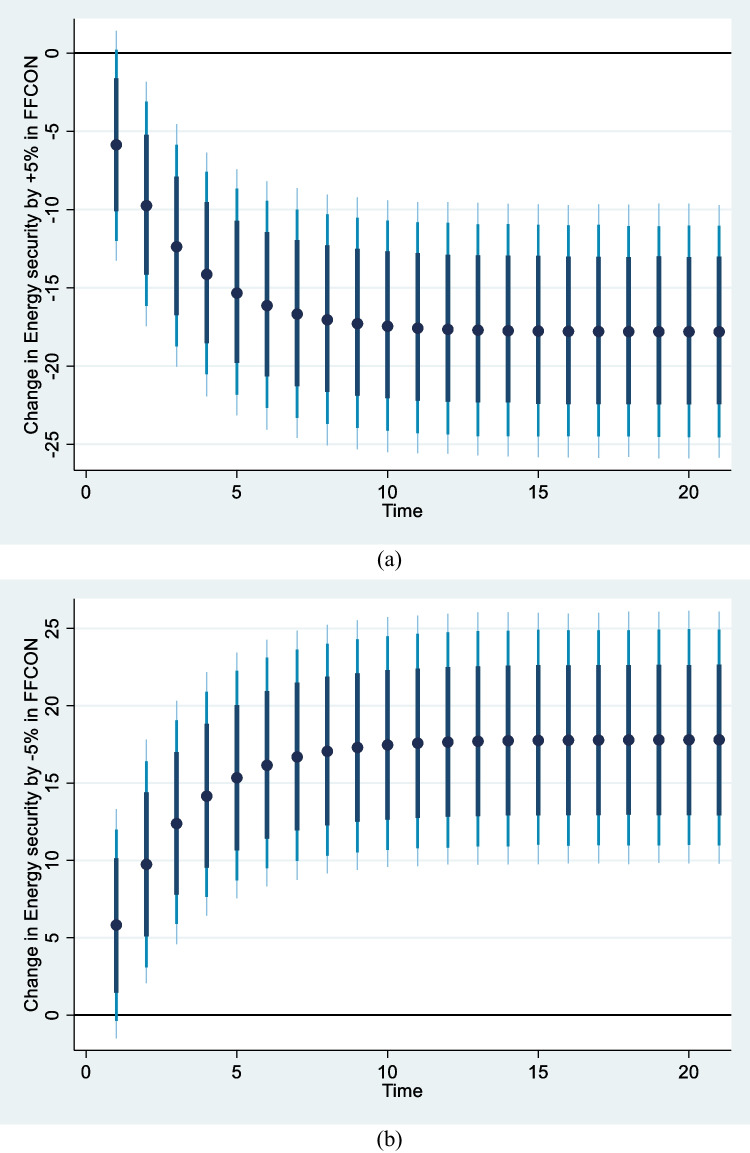
Response of energy security because of 10% **a** increase and **b** decrease in fossil fuel consumption

**Fig. 7:**
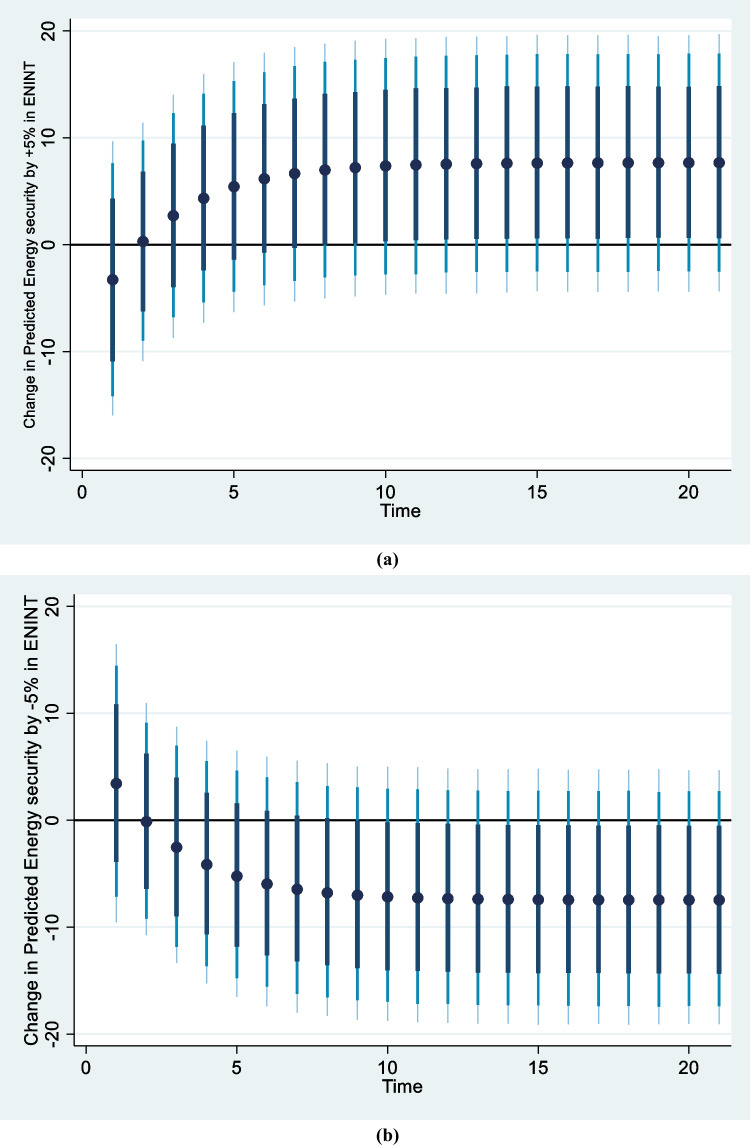
Response of energy security to a 5% **a** increase and **b** decrease in energy intensity

**Fig. 8:**
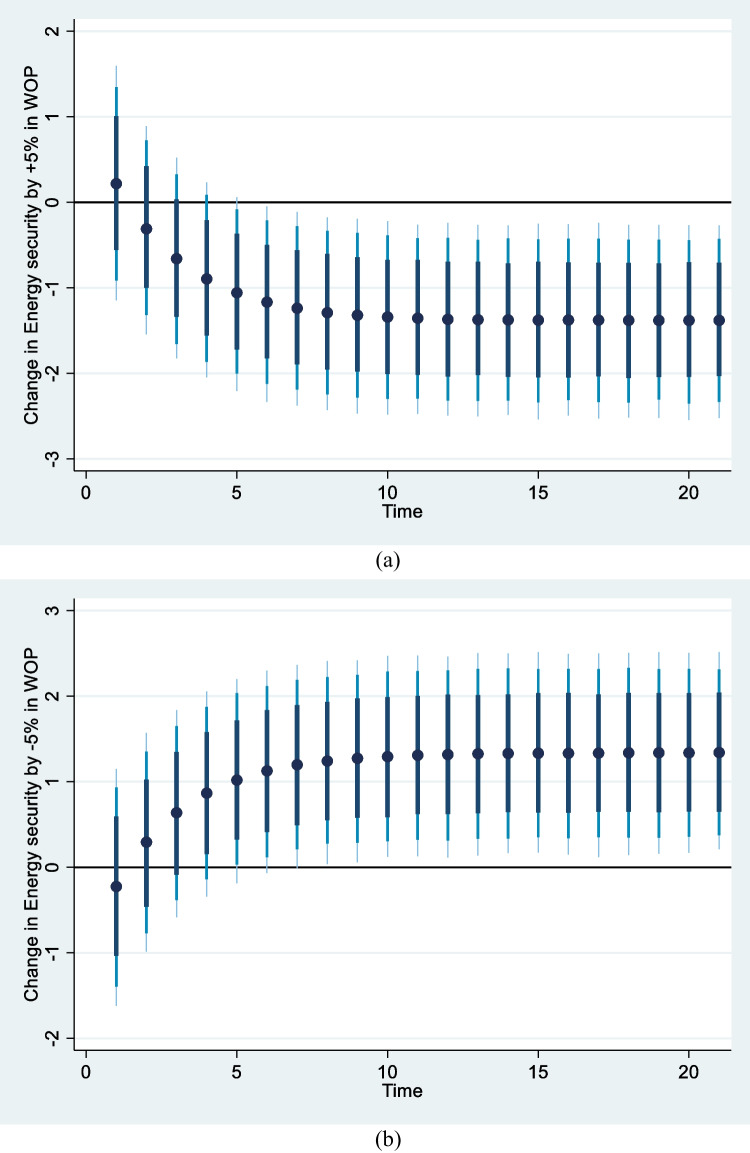
Response of energy security to a 12% **a** increase and **b** decrease in global oil prices

The responses of energy security to a change in one of the explanatory variables are presented in Figs. 4, 5, 6, 7, and 8 in the Appendix, while other variables are constant. The results of these figures show that the impact of a 5% increase (or decrease) in economic growth (RGDP) increases (or decreases) energy security by about 10% in the long run (Fig. 4). The response of energy security to a 5% increase (or decrease) in renewable energy consumption increases (or decreases) energy security in the long run (Fig. 5).

The response of energy security to a 5% increase (decrease) in fossil fuel consumption is associated with a decrease (increase) in energy security in the long run (Fig. 6). When energy intensity increases (or decreases) by 5% (Fig. 7), we can see that the energy security of the country improves (or declines) by about 7% during the first 7 years and then continues with this range in the long run. Finally, energy security decreases and increases in response to a 5% increase and decrease in global oil prices, respectively (Fig. 8).

## Conclusions and policy implications

This study investigated the impacts of key variables on energy security in an oil-importing country, i.e., New Zealand. It used dynamic ARDL simulations and vector autoregression (VAR) methods on data for New Zealand from 1978 to 2021.

The study results show that there are long-run cointegration relationships between energy security, economic growth (RGDP), renewable and fossil fuel consumption, and energy intensity. Economic growth has a positive and significant impact on energy security in the short and long run. This may be due to the low reliance of NZ agriculture on fossil fuel consumption, as agriculture is the primary driver of NZ’s economic growth. The results of the dynamic simulations model support this outcome and show that if economic growth increases by 5%, energy security increases during the first 6 years by 10% and then continues at this level. Energy intensity also improves energy security in the short and long run because of the improvement of energy efficiency over time.

When energy intensity improves by 5%, energy security increases by about 8% during the first 7 years and continues at this rate in the long run. Renewable energy consumption as a proxy to produce renewable energy increases energy security because the country is more dependent on its internal energy resources and can reduce its dependency on external energy sources. In the simulation model, the response to a 5% increase in renewable energy consumption is an increase in energy security of 7%. Fossil fuel consumption reduces energy security, and its impact is significant in both the short and long run.

The response of a 5% increase in fossil fuel consumption reduces energy security by 18%. Finally, global oil prices, by increasing the prices of energy resources and decreasing their affordability, reduce energy security. Energy security decreases (or increases) by 1.5% in response to a 5% increase (or decrease) in global oil prices. Finally, a unidirectional causality exists from real GDP, fossil fuel consumption, and renewable energy use to energy security. The causal link between energy intensity and energy security is bidirectional.

The results of this study suggest that to minimize the harmful effects of sudden crude oil price fluctuations, it is important to maintain strategic petroleum reserve stocks for at least 1 year. This is because many oil shocks are short run, and most economies can adjust themselves to the changes that happen in the oil market. As the transport sector is the main consumer of petroleum products that the country imports from other countries, one of the strategic things that the country can do to improve its energy security is the production of biofuels to reduce its dependency on imported petroleum products. On the other hand, producing other renewable energies, such as electricity from hydropower plants and geothermal, is necessary to support other requirements of the country. This is because of the existence of the neutrality hypothesis, which means that the government can introduce measures to improve energy efficiency in the country without fear of harming economic growth. But the caution is for fossil fuel policies that affect economic growth. Enhancing efficient technologies through a large scale of green finance and green investment projects can improve New Zealand’s energy security status.

## Data Availability

The data will be available upon the request.

## Appendix

**Table 10:** Error correction model: ARDL (3, 4, 4, 3, 2, 3, 2), with a new index for energy security

Variable	Coefficient	Std. error	*t*-statistic	Prob
RETES(− 1)*	− 1.046	0.274	− 3.816	0.002
RGDP(− 1)	1.693	0.391	4.335	0.001
RCON(− 1)	0.097	0.180	0.539	0.600
FCON(− 1)	− 1.705	0.364	− 4.682	0.001
ENINT(− 1)	0.831	0.264	3.153	0.008
WOP(− 1)	− 0.068	0.040	− 1.710	0.113
FINDEV(− 1)	0.008	0.008	1.078	0.302
*C*	− 7.305	2.028	− 3.602	0.004
*D*(RETES(− 1))	0.764	0.395	1.936	0.077
*D*(RETES(− 2))	0.297	0.202	1.468	0.168
*D*(RGDP)	1.162	0.578	2.010	0.067
*D*(RGDP(− 1))	− 1.044	0.435	− 2.402	0.033
*D*(RGDP(− 2))	− 1.039	0.425	− 2.445	0.031
*D*(RGDP(− 3))	− 1.443	0.335	− 4.308	0.001
*D*(RCON)	0.215	0.157	1.369	0.196
*D*(RCON(− 1))	− 0.188	0.258	− 0.730	0.480
*D*(RCON(− 2))	− 0.172	0.183	− 0.941	0.365
*D*(RCON(− 3))	0.465	0.163	2.851	0.015
*D*(FCON)	− 1.015	0.183	− 5.533	0.000
*D*(FCON(− 1))	1.541	0.402	3.832	0.002
*D*(FCON(− 2))	0.588	0.217	2.711	0.019
*D*(ENINT)	0.274	0.219	1.249	0.235
*D*(ENINT(− 1))	− 0.764	0.404	− 1.889	0.083
*D*(WOP)	− 0.032	0.023	− 1.376	0.194
*D*(WOP(− 1))	0.000	0.026	0.017	0.987
*D*(WOP(− 2))	− 0.034	0.019	− 1.785	0.100
*D*(FINDEV)	− 0.001	0.003	− 0.392	0.702
*D*(FINDEV(− 1))	− 0.007	0.004	− 1.863	0.087
*R*-squared	0.958	Mean dependent var		0.003
Adjusted *R*-squared	0.864	S.D. dependent var		0.050
S.E. of regression	0.018	Akaike info criterion		− 4.972
Sum squared resid	0.004	Schwarz criterion		− 3.790
Log likelihood	127.446	Hannan-Quinn criter		− 4.545
*F*-statistic	10.200	Durbin-Watson stat		2.136
Prob (*F*-statistic)	0.000			

**p*-values are incompatible with *t*-bounds distribution

**Table 11:** Bayesian VAR estimates

	ESEC	RGDP	RCON	FCON	ENINT	WOP	FINDEV
ESECIN(− 1)	0.918	0.012	− 0.017	− 0.006	0.006	0.026	− 0.076
	(0.083)	(0.016)	(0.021)	(0.026)	(0.022)	(0.151)	(0.942)
ESECIN(− 2)	− 0.006	0.000	− 0.005	− 0.006	− 0.003	− 0.004	− 0.119
	(0.048)	(0.009)	(0.012)	(0.015)	(0.013)	(0.087)	(0.538)
ESECIN(− 3)	− 0.006	0.000	− 0.001	− 0.001	− 0.001	− 0.006	− 0.011
	(0.033)	(0.006)	(0.008)	(0.010)	(0.009)	(0.059)	(0.368)
RGDP(− 1)	0.113	0.989	0.007	− 0.015	0.014	0.006	0.332
	(0.344)	(0.066)	(0.086)	(0.110)	(0.091)	(0.631)	(3.924)
RGDP(− 2)	− 0.011	− 0.004	− 0.002	0.000	− 0.008	− 0.061	− 0.274
	(0.239)	(0.046)	(0.059)	(0.076)	(0.063)	(0.438)	(2.726)
RGDP(− 3)	0.005	− 0.004	0.005	0.004	0.002	0.034	− 0.064
	(0.167)	(0.032)	(0.041)	(0.053)	(0.044)	(0.305)	(1.899)
ENINT(− 1)	0.068	0.026	0.954	0.037	0.000	− 0.177	− 0.914
	(0.329)	(0.063)	(0.082)	(0.105)	(0.087)	(0.604)	(3.754)
ENINT(− 2)	0.050	0.005	0.005	0.022	0.014	0.066	0.552
	(0.190)	(0.036)	(0.048)	(0.061)	(0.050)	(0.349)	(2.167)
ENINT(− 3)	0.018	0.004	− 0.005	0.004	0.001	0.023	− 0.088
	(0.130)	(0.025)	(0.033)	(0.042)	(0.034)	(0.238)	(1.482)
RECON(− 1)	0.032	0.012	− 0.041	0.962	− 0.017	0.275	− 0.417
	(0.233)	(0.044)	(0.058)	(0.075)	(0.062)	(0.428)	(2.659)
RECON(− 2)	− 0.004	0.000	0.001	− 0.004	0.002	0.047	0.327
	(0.146)	(0.028)	(0.036)	(0.047)	(0.039)	(0.267)	(1.659)
RECON(− 3)	− 0.010	0.001	0.000	− 0.001	− 0.002	0.027	− 0.136
	(0.100)	(0.019)	(0.025)	(0.032)	(0.027)	(0.184)	(1.142)
FFCON(− 1)	0.059	0.022	− 0.034	0.030	0.942	− 0.276	− 0.107
	(0.296)	(0.056)	(0.074)	(0.095)	(0.079)	(0.543)	(3.377)
FFCON(− 2)	0.027	0.003	0.005	0.031	0.002	0.079	0.126
	(0.177)	(0.034)	(0.044)	(0.057)	(0.047)	(0.324)	(2.014)
FFCON(− 3)	0.003	0.000	− 0.002	0.006	− 0.003	0.058	− 0.120
	(0.122)	(0.023)	(0.030)	(0.039)	(0.032)	(0.223)	(1.385)
WOP(− 1)	− 0.017	0.002	− 0.003	− 0.012	− 0.003	0.916	0.010
	(0.047)	(0.009)	(0.012)	(0.015)	(0.012)	(0.086)	(0.532)
WOP(− 2)	0.003	0.001	0.000	− 0.001	− 0.001	− 0.017	− 0.001
	(0.026)	(0.005)	(0.006)	(0.008)	(0.007)	(0.048)	(0.297)
WOP(− 3)	0.001	0.001	0.000	0.000	0.000	− 0.004	− 0.040
	(0.018)	(0.003)	(0.004)	(0.006)	(0.005)	(0.033)	(0.201)
FINDEV(− 1)	− 0.001	0.000	0.000	− 0.001	0.000	0.001	0.869
	(0.008)	(0.002)	(0.002)	(0.003)	(0.002)	(0.015)	(0.091)
FINDEV(− 2)	0.000	0.000	0.000	0.000	0.000	− 0.001	0.000
	(0.004)	(0.001)	(0.001)	(0.001)	(0.001)	(0.008)	(0.049)
FINDEV(− 3)	0.000	0.000	0.000	0.000	0.000	0.000	0.003
	(0.003)	(0.001)	(0.001)	(0.001)	(0.001)	(0.005)	(0.033)
*C*	− 1.696	− 0.023	0.323	− 0.081	0.315	0.350	2.852
	(2.908)	(0.554)	(0.723)	(0.931)	(0.769)	(5.328)	(33.124)
Adj. *R*-squared	0.369	0.985	0.885	0.964	0.928	0.672	− 1.571
*F*-statistic	2.112	123.397	15.620	51.941	25.611	4.901	− 0.164

Standard errors in ()

## Declarations

### Human or animal participants

This article does not contain any studies with human participants or animals performed by the author.

The author of this research confirms that the submitted work is original and has not been published elsewhere in any form or language (partially or in full).
